# Schizotypal Traits are Associated with Decreased Functional Connectivity between Medial Prefrontal Cortex and Cerebellum in a Non-clinical Sample

**DOI:** 10.1192/j.eurpsy.2022.558

**Published:** 2022-09-01

**Authors:** Y. Panikratova, E. Abdullina, E. Pechenkova, I. Lebedeva

**Affiliations:** 1 Mental Health Research Center, Laboratory Of Neuroimaging And Multimodal Analysis, Moscow, Russian Federation; 2 Higher School of Economics, Laboratory For Cognitive Research, Moscow, Russian Federation

**Keywords:** resting-state fMRI, schizotypy, SPQ-74

## Abstract

**Introduction:**

Schizotypy is associated with increased vulnerability to schizophrenia spectrum disorders. Therefore, investigation of its brain correlates seems prominent for better understanding of schizophrenia-spectrum continuum as well as for development of biological treatments for schizotypal personality disorder. Functional alterations of prefrontal cortex (PFC) and their associations with clinical symptoms are well-known to exist in schizophrenia. However, their relevance to schizotypy remains unclear.

**Objectives:**

The aim of the study was to check for associations between schizotypal traits in a non-clinical sample and whole-brain functional connectivity (FC) of lateral as well as medial PFC (lPFC and mPFC, respectively).

**Methods:**

Eighty-two healthy individuals (52 females, mean age 24.8±5.48) filled out the Schizotypal Personality Questionnaire (SPQ-74) and underwent resting-state fMRI (3T). Seeds in lPFC and mPFC were taken from frontoparietal and default mode networks (atlas by Yeo et al., 2011). We analyzed correlations between four schizotypal factors (cognitive/perceptual, paranoid, negative, and disorganization; Stefanis et al., 2004) and whole-brain FC of the seeds (statistical threshold: *p*<.001 voxelwise; *p[FDR]*<.05 clusterwise).

**Results:**

Cognitive/perceptual factor (‘Odd beliefs/magical thinking’ and ‘Unusual perceptual experiences’ SPQ-74 subscales) is negatively correlated to FC of bilateral mPFC with a cluster in the right cerebellum (Crus 1, 2).

**Conclusions:**

Prefrontal-cerebellar dysconnectivity may be one of the neurobiological factors underlying positive-symptoms-like schizotypal traits in non-clinical subjects. To some extent, it coincides with the data on associations between functional features of these brain structures and positive symptoms in schizophrenia (Pinheiro et al., 2021; Goghari et al., 2010).

**Disclosure:**

The study was supported by RFBR Grant 20-013-00748.

